# Robust Sliding Mode Control of PMSM Based on Rapid Nonlinear Tracking Differentiator and Disturbance Observer

**DOI:** 10.3390/s18041031

**Published:** 2018-03-29

**Authors:** Zhanmin Zhou, Bao Zhang, Dapeng Mao

**Affiliations:** 1 Changchun Institute of Optics, Fine Mechanics and Physics, Chinese Academy of Sciences, Changchun 130033, China; zhangb@ciomp.ac.cn (B.Z.); mdp_ciomp@126.com (D.M.); 2 University of Chinese Academy of Sciences, No.19, Yuquan Rd., Beijing 100049, China

**Keywords:** permanent magnet synchronous motor, inertia stability, sliding mode control, nonlinear tracking differentiator, disturbance observer, robust

## Abstract

Torque ripples caused by cogging torque, flux harmonics, and current measurement error seriously restrict the application of a permanent magnet synchronous motor (PMSM), which has been paid more and more attention for the use in inertial stabilized platforms. Sliding mode control (SMC), in parallel with the classical proportional integral (PI) controller, has a high advantage to suppress the torque ripples as its invariance to disturbances. However, since the high switching gain tends to cause chattering and it requires derivative of signals which is not readily obtainable without an acceleration signal sensor. Therefore, this paper proposes a robust SMC scheme based on a rapid nonlinear tracking differentiator (NTD) and a disturbance observer (DOB) to further improve the performance of the SMC. The NTD is employed to providing the derivative of the signal, and the DOB is utilized to estimate the system lumped disturbances, including parameter variations and external disturbances. On the one hand, DOB can compensate the robust SMC speed controller, it can reduce the chattering of SMC on the other hand. Experiments were carried out on an ARM and DSP-based platform. The obtained experimental results demonstrate that the robust SMC scheme has an improved performance with inertia stability and it exhibits a satisfactory anti-disturbance performance compared to the traditional methods.

## 1. Introduction

As its attractive characteristics such as efficiency, power density, torque-to-inertia ratio, reliability, etc., the application of permanent magnet synchronous motors (PMSM) on inertial stabilized platforms have been paid more and more attention. Usually, an airborne inertial stabilization photoelectric platform is equipped with visible or infrared cameras, and if we want to obtain stable and clear images, the stability of the platform must be high enough. However, due to the influence of inherent factors, such as the cogging torque of the permanent magnet synchronous motor, the torque ripples caused by the motor during operation seriously affects the performance of the motor. The torque ripples will also produce noise when the servo system is running at high speed and even cause the system to vibrate mechanically at low speed [1,2,3].

There are several ways to weaken the torque ripple from the optimization design of the motor, and it is the most effective means, such as skew of the stator lamination stack or rotor magnetization, skewing and fractional slot pitch windings, increasing the number of phases, dummy slots, and dummy teeth, optimization of the air gap flux distribution, and the magnet pole arc width and position, etc. [4]. However, optimizing the design of the motor usually makes it difficult to manufacture, and it will increase the cost significantly.

Active control is another method to reduce the torque ripple by changing the input current or voltage to achieve the desired torque output. Many scholars have performed a great deal of work in this area and have made many achievements. Thus, we can only achieve the purpose by changing the control strategy instead of the motor itself. No matter what way the PMSM is controlled, the controller is needed, so there is no extra cost. Additionally, by active control techniques, the design and manufacturing cycle of the motor can also be saved [5,6].

In previous works, Hung designed a torque ripple suppression strategy based on position compensation [7]. However, the exact correspondence between the torque ripple and the position must be known if this method is used, otherwise the result may be worse. In fact, this is an open-loop compensation strategy. For closed-loop control, an effective way is to use torque transducer, but this will make the structure of control system more complex and increase the cost. The effect of the traditional PI control on the torque ripple caused by the mechanical parts (e.g., cogging torque and load oscillations) is also not satisfactory. Iterative learning control is used in [8,9,10], it gradually eliminates periodic torque ripples by iteration. This is indeed an effective way to eliminate the periodic torque ripples of the PMSM, but it is not suitable on the optoelectronic stabilization platform. Since the motion of the aerial photoelectricity platform is usually random and works at a low speed.

SMC is a popular nonlinear method for PMSM control [11,12,13,14,15]. As the sliding mode can be designed and independent of the object parameters and disturbances, SMC has the advantages of quick response, insensitivity to parameter changes and disturbances, no on-line identification, and simple physical realization. In particular, it is a very attractive aspect of the invariance to the disturbance [16]. Sliding mode variable structure control has also been successfully applied in many fields [17,18,19,20,21]. However, the invariance of the sliding mode variable structure control to the parameter perturbation and external disturbance of the system is changed by the high-frequency jitter of the control quantity.

Therefore, in this paper, on the basis of the design of a robust sliding mode controller, a disturbance observer is introduced to reduce the switching gain, so that the buffeting effect of the system can be weaken. The two-order derivative of the signal is usually needed in the sliding mode controller, but the use of speed sensors or accelerometers will make the system complex and increase the cost significantly. The traditional differential method is divided into two main types: one is to calculate the change of signal in unit time, or to calculate the time used by the unit change quantity. However, the traditional differential method is not ideal. Although the Kalman filter and the dual-sampling-rate observer are presented, they are also limited in practice for the model needs to be known, while we cannot always obtain the accurate model of the system [22,23,24]. A fast-nonlinear tracking differentiator for practical application of engineering is discussed in [25]. The problem of the phase lags of the differentiator has been considered and a feedforward is used to improve the differential estimate by extends the traditional structure and provides an additional freedom for the design of NTD [26]. It improves the accuracy of the differential estimate compared with the traditional method and is suitable for engineering practice.

This paper is organized as follows. In the second part, the mathematical model of PMSM is established. The scheme of a robust SMC based on rapid NTD and DOB is introduced in three sections in the third part, where the convergence of the method and the control structure of the whole system are also given in this part. Section 4 introduces the construction of the experimental platform for PMSM. In Section 5, the effectiveness of the proposed robust SMC scheme is proved by experiments, and the results are discussed. The last part of the article gives the conclusion.

## 2. Mathematical Model of PMSM

**Figure 1:**
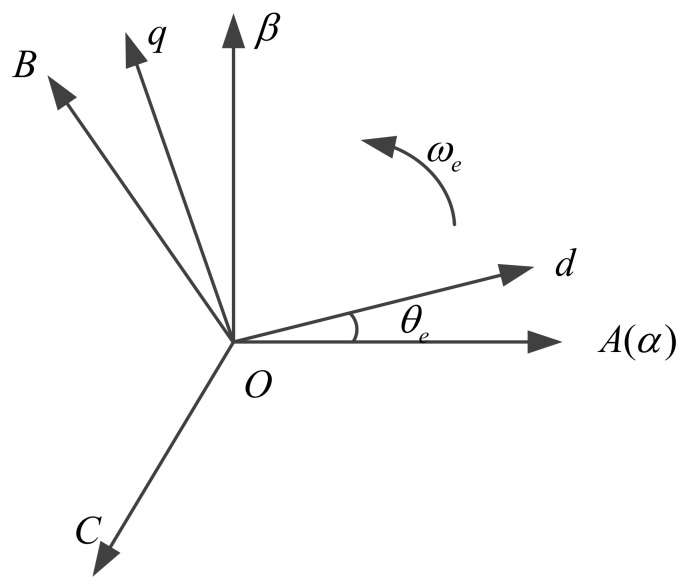
Relationship between the various coordinate systems.

In order to simplify the mathematical model of three phase PMSM in the natural coordinate system, the coordinate transformation usually includes static coordinate transformation (Clark transformation) and synchronous rotation coordinate transformation (Park transformation). The relationship between them is shown in Figure 1, in which ABC is a natural coordinate system, α−β, is a stationary coordinate system, and d−q is a synchronous rotating coordinate system.

The coordinate transformation of the natural coordinate system ABC to the stationary coordinate system α−β is the Clark transformation. According to the relationship between the various coordinate systems shown Figure 1, the formula is as follows:

(1) [fα fβ f0]T=T[fA fB fC]T3s/2s

Among them, f is the variable of motor voltage, current or magnetic chain, and $T_{3s/2s}$ is the coordinate transformation matrix, which can be expressed as follows:

(2) $$T_{3s/2s}=\frac{2}{3}\left[\begin{array}{ccc} 1 & -\frac{1}{2} & -\frac{1}{2}\\ 0 & \frac{\sqrt{3}}{2} & -\frac{\sqrt{3}}{2}\\ \frac{\sqrt{2}}{2} & \frac{\sqrt{2}}{2} & \frac{\sqrt{2}}{2} \end{array}\right]$$

where the coefficient 2/3 is obtained according to the amplitude as the constraint condition. When the power constant is used as the constraint condition, the coefficient becomes $\sqrt{2/3}$.

The coordinate transformation of the stationary coordinate system α−β to the synchronous rotating coordinate system d−q is the Park transformation:

(3) $${\left[f_{d}\;f_{q}\right]}^{T}=T_{2s/2r}{\left[f_{\alpha }\;f_{\beta }\right]}^{T}$$

where $T_{2s/2r}$ is the coordinate transformation matrix, which can be expressed as follows:

(4) $$T_{2s/2r}=\left[\begin{array}{cc} \cos\theta_{e} & \sin\theta_{e}\\ -\sin\theta_{e} & \cos\theta_{e} \end{array}\right]$$

According to the above relationship, the transformation relationship between the transformation of the natural coordinate system ABC to the synchronous rotating coordinate system d−q can be obtained:

(5) $${\left[f_{d}\;f_{q}\;f_{0}\right]}^{T}=T_{3s/2r}{\left[f_{A}\;f_{B}\;f_{c}\right]}^{T}$$

$T_{3s/2r}$ is the coordinate transformation matrix, which can be expressed as follows:

(6) $$T_{3s/2r}=T_{2s/2r}\times T_{3s/2s}^{\prime }=\frac{2}{3}\left[\begin{array}{ccc} \begin{array}{l} \cos\theta \\ -\sin\theta_{e} \end{array} & \begin{array}{l} \cos(\theta_{e}-2\pi /3)\\ -\sin(\theta_{e}-2\pi /3) \end{array} & \begin{array}{l} \cos(\theta_{e}+2\pi /3)\\ -\sin(\theta_{e}+2\pi /3) \end{array} \end{array}\right]$$

where ${T^{\prime }}_{3s/2s}$ is the top two rows of $T_{3s/2s}$.

It is necessary to point out that for a three-phase symmetric system, the zero-sequence component $f_{0}$ can be ignored when calculating.

In order to facilitate the design of the controller, we choose the mathematical model under the synchronous rotating coordinate system d−q [27,28]. The stator voltage equation is as follows:

(7) $$\left\{ \begin{array}{l} u_{d}=Ri_{d}+\frac{d\psi_{d}}{dt}-\omega_{e}\psi_{q}\\ u_{q}=Ri_{q}+\frac{d\psi_{q}}{dt}+\omega_{e}\psi_{d} \end{array}\right.$$

The stator flux equation is as follows:

(8) $$\left\{ \begin{array}{l} \psi_{d}=L_{d}i_{d}+\psi_{f}\\ \psi_{q}=L_{q}i_{q} \end{array}\right.$$

Then we can get a new stator voltage equation:

(9) $$\left\{ \begin{array}{l} u_{d}=Ri_{d}+L_{d}\frac{di_{d}}{dt}-\omega_{e}L_{q}i_{q}\\ u_{q}=Ri_{q}+L_{q}\frac{di_{q}}{dt}+\omega_{e}(L_{d}i_{d}+\psi_{f}) \end{array}\right.$$

where $u_{d}$ and $u_{q}$ are the stator voltage along the d and q axes, respectively, $i_{d}$ and $i_{q}$ are the stator current along the d and q axes, respectively, R is the stator resistance, $\psi_{d}$ and $\psi_{q}$ are the stator flux linkages along the d and q axes, respectively, $\omega_{e}$ is the electrical angular speed, $L_{d}$ and $L_{q}$ are the inductances along the d and q axes, respectively, and $\psi_{f}$ are the flux linkages of the permanent magnet.

According to the Equation (9), we can find that the mathematical model of PMSM is fully decoupled. Then we can get the electromagnetic torque equation:

(10) $$T_{e}=\frac{3}{2}pi_{q}[i_{d}(L_{d}-L_{q})+\psi_{f}]$$

Equations (7)–(10) are the mathematical model for the built-in PMSM, and for the surface mounted PMSM, the stator inductance $L_{d}=L_{q}$. Then we can obtain:

(11) $$T_{e}=\frac{3}{2}pi_{q}\psi_{f}=\;K_{t}i_{q}$$

where $T_{e}$ is the electromagnetic torque, p is the number of pole pairs and $K_{t}$ is the torque coefficient. The equation of PMSM dynamic is:

(12) $$J\frac{d\omega_{m}}{dt}=T_{e}-T_{L}-B\omega_{e}$$

where J is the inertia, $\omega_{m}$ is the mechanical angular speed, $T_{L}$ is the load torque, and B is the viscous frictional coefficient.

In fact, the above model is only obtained under ideal conditions. Parasitic torque pulsations exist in PMSM due to the non-sinusoidal flux density distribution around the air gap, errors in current measurements, and variable magnetic reluctance of the air gap due to the stator slots [8]. The speed of the motor will oscillate as a result, especially at low operating speeds. Therefore, to reduce the speed ripples, an appropriate control strategy is needed to minimize the torque ripple.

## 3. Design of Robust Sliding Mode Control

### 3.1. Robust Sliding Mode Control

SMC is essentially a kind of nonlinear control method, and its nonlinearity expressed as the discontinuity of the control variables. The difference between SMC and other control strategy is that the “structure” is not fixed. SMC can change according to the current state (such as the error and its derivative) of the system in the dynamic process, forcing the system to move in accordance with the state trajectory of a predetermined “sliding mode”. Following is the robust SMC strategy used in this paper.

According to Equation (12), we can obtain the mathematical model of the PMSM:

(13) $$J{\ddot{\theta }}_{m}=K_{t}i_{q}+D$$

where D is the system lumped disturbance, and $\theta_{m}$ is the mechanical angular. In order to facilitate the subsequent deduction, we use B to represent $J/K_{t}$, to represent $D/K_{t}$, and remove the subscripts of $\theta_{m}$:

(14) $$B\ddot{\theta }=i_{d}+d$$

Without loss of generality, suppose that the parameters B and d are bounded, $|B|\leq \Delta_{1},\;|d|\leq \Delta_{2}$.

The control objective is $e=\theta -\theta_{d}=0$, and we select the following sliding mode function [29]:

(15) $$\sigma =\dot{e}+\alpha s(e)$$

where α>0, and s(e) is the saturation error function, we define that:

(16) $$\rho (e)=\sqrt{c^{2}+e^{2}}-|c|$$

of which c is an arbitrary constant:

(17) $$s(e)=\frac{d\rho }{de}=\frac{e}{\sqrt{c^{2}+e^{2}}}$$

Then we can get following properties of ρ(e) and s(e):

(18) $$\left\{ \begin{array}{l} \dot{\rho }(e)=s(e)\dot{e}\\ \dot{s}(e)=\frac{ds(e)}{de}\dot{e}=\frac{c^{2}\dot{e}}{\sqrt{{\left(c^{2}+e^{2}\right)}^{3}}} \end{array}\right.$$

According to the above formula, we can obtain:

(19) $$\dot{\theta }=\dot{e}+{\dot{\theta }}_{d}$$

and:

(20) $$\ddot{\theta }={\ddot{\theta }}_{d}+\ddot{e}=\dot{\sigma }+{\ddot{\theta }}_{d}-\alpha \dot{s}(e)$$

Multiplied by B on both sides of Equation (20) yields:

(21) $$B\dot{\sigma }=i_{d}-B{\ddot{\theta }}_{d}+B\alpha \dot{s}(e)+d$$

Then we can get the following sliding mode controller:

(22) $$i_{d}=-k_{p}e-k_{v}\dot{e}-k_{t}\sigma -\eta \frac{\sigma }{\left|\sigma \right|},\;k_{p},k_{v},k_{t}>0,\eta >0$$

The following is a proof of its large scale asymptotic stability.

According to Equations (21) and (22):

(23) $$B\dot{\sigma }=-k_{p}e-k_{v}\dot{e}-k_{t}\sigma -\eta \frac{\sigma }{\left|\sigma \right|}+B\alpha \dot{s}(e)-B{\ddot{\theta }}_{d}+d$$

Multiplied by σ on both sides of Equation (23) yields:

(24) $$\sigma B\dot{\sigma }=-\beta -\eta |\sigma |-\sigma (B{\ddot{\theta }}_{d}-d)$$

where:

(25) $$\begin{array}{l} \beta =\sigma \left[k_{p}e+k_{v}\dot{e}+k_{t}\sigma -B\alpha \dot{s}(e)\right]\\ \text{ߓ}=k_{p}e\dot{e}+(k_{v}+k_{t}){\dot{e}}^{2}-\alpha B\dot{s}(e)\dot{e}+\alpha (k_{v}+2k_{t})s(e)\dot{e}\\ \text{ߓ}+\alpha k_{p}es(e)+\alpha^{2}k_{t}s^{2}(e)-\alpha^{2}Bs(e)\dot{s}(e) \end{array}$$

According to Equation (18), the positive definite functions $v_{1}$ and $v_{2}$ can be defined as follows:

(26) $$\left\{ \begin{array}{l} v_{1}=\frac{1}{2}k_{p}e^{2}+\alpha (k_{v}+2k_{t})\rho (e)-\frac{1}{2}\alpha^{2}Bs^{2}(e)\\ v_{2}=(k_{v}+k_{t}){\dot{e}}^{2}+\alpha k_{p}es(e)-\alpha B\dot{s}(e)\dot{e}+\alpha^{2}k_{t}s^{2}(e) \end{array}\right.$$

where $k_{p}>\frac{1}{c^{2}}\alpha^{2}\Delta_{1},\;k_{v}+k_{t}>\frac{1}{\left|c\right|}\alpha \Delta_{1}$, then β can be expressed as:

(27) $$\beta ={\dot{v}}_{1}+v_{2}$$

Further we can get:

(28) $$\left\{ \begin{array}{l} k_{p}e^{2}>\frac{e^{2}}{c^{2}}\alpha^{2}\Delta_{1}>\frac{e^{2}}{c^{2}}\alpha^{2}B>\frac{e^{2}}{c^{2}+e^{2}}\alpha^{2}B=\alpha^{2}Bs^{2}(e)\\ (k_{v}+k_{t}){\dot{e}}^{2}>\frac{1}{\left|c\right|}\alpha \Delta_{1}{\dot{e}}^{2}>\frac{c^{2}\dot{e}}{\sqrt{{\left(c^{2}+e^{2}\right)}^{3}}}\alpha B\dot{e}=\alpha B\dot{s}(e)\dot{e} \end{array}\right.$$

At last, if Lyapunov function is selected as follows:

(29) $$V=\frac{1}{2}B\sigma^{2}+v_{1}$$

then:

(30) $$\begin{array}{ll} \dot{V} & =\sigma B\dot{\sigma }+{\dot{v}}_{1}=-\beta -\eta |\sigma |-\sigma (B{\ddot{\theta }}_{d}-d)+{\dot{v}}_{1}\\ & =-v_{2}-|\sigma |[\eta +\frac{\sigma }{\left|\sigma \right|}(B{\ddot{\theta }}_{d}-d)] \end{array}$$

Therefore, if $|B|\leq \Delta_{1},\;|d|\leq \Delta_{2}$ and $|{\ddot{\theta }}_{d}|\leq \Delta_{a}$, the following result can be obtained:

(31) $$\dot{V}=-v_{2}-|\sigma |[\eta -(\Delta_{1}\Delta_{a}+\Delta_{2})]\leq -v_{2},\forall \eta >\Delta_{1}\Delta_{a}+\Delta_{2}$$

As $v_{2}$ is positive definite, so $\dot{V}$ is negative definite. Therefore, the stability of the system and the gradual stability of large range can be guaranteed by the sliding mode controller $i_{d}$.

Further using the boundary layer method to improve the control law yields:

(32) $$u_{c}=-k_{p}e-k_{v}\dot{e}-k_{t}\sigma -\eta sat(\frac{\sigma }{\psi })$$

where:

(33) $$sat(\frac{\sigma }{\psi })=\left\{ \begin{array}{l} -1\text{ߓ}\sigma <-\psi \\ \frac{\sigma }{\psi }\text{ߓ}-\psi \leq \sigma \leq \psi \\ 1\text{ߓ}\sigma >\psi \end{array}\right.$$

**Figure 2:**
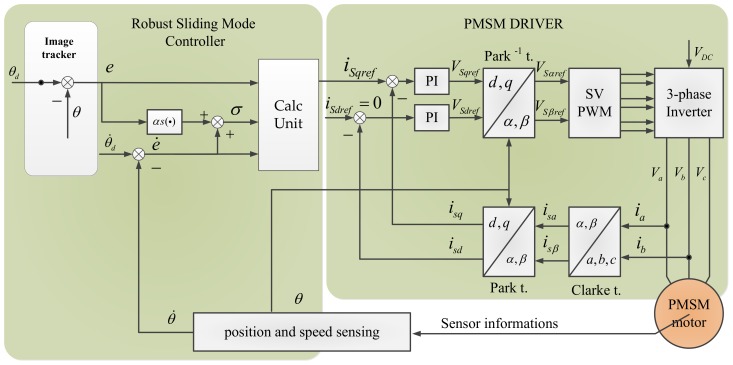
Block diagram of robust sliding mode control.

The overall block diagram of the robust sliding mode control is shown in Figure 2. The field-oriented control (FOC) method is utilized to control the PMSM. The robust sliding mode controller is employed as the speed controller to generate the q-axis reference current $i_{sqref}$. In order to obtain maximum electromagnetic torque, $i_{sqref}$ is always assigned to zero. e is measured by an image tracker, $\dot{\theta }$ and θ are measured by a gyroscope and an encoder, respectively.

### 3.2. Disturbance Observer

**Figure 3:**
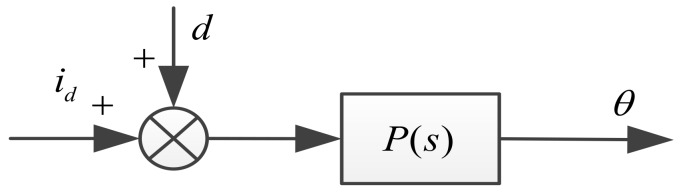
Block diagram of Equation (14).

**Figure 4:**
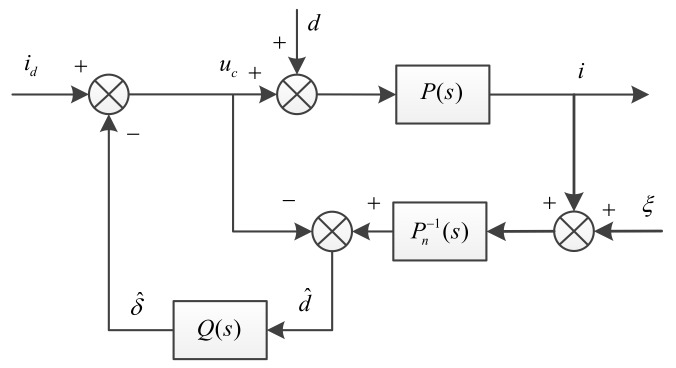
Typical disturbance observer.

In the above proof we notice that the switching gain η needs to be greater than $\Delta_{1}\Delta_{a}+\Delta_{2}$. Generally, $\Delta_{1}$ and $\Delta_{a}$ can be obtained or estimated from actual system, but we cannot obtain the exact values of $\Delta_{2}$, because it is not only related to the parameters of the motor itself, but also many factors, such as the tightness of the mechanical installation and the disturbance caused by the cable, and so on. In order to make the system stable, we usually use a high gain, but excessive gain will easily cause the chattering of the system. Thus, a natural idea is that if we observe the disturbance and compensate it in the system, the buffeting effect of the system can be greatly reduced. Equation (14) can be drawn as Figure 3.

where $P(s)=\frac{1}{Bs^{2}}$, a typical DOB based on the nominal model is shown in Figure 4 [30].

where $P_{n}(s)$ is the nominal model of practical system, and $P_{n}^{-1}(s)$ is its inverse model, Q(s) is a low-pass filter, and ξ is the measurement noise.

(34) $$\begin{array}{ll} \hat{\delta } & \approx Q(s)\{[(u_{c}+d)P(s)]P_{n}^{-1}(s)-u_{c}\}\\ & =Q(s)\{[(u_{c}+d)P(s)]P_{n}^{-1}(s)-u_{c}\}\\ & =Q(s)d \end{array}$$

**Figure 5:**
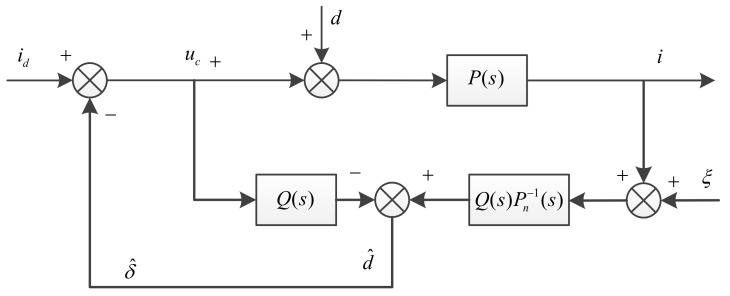
Disturbance observer after structural transformation.

This structure uses the inverse of the nominal model to estimate the disturbance, but the relative order of the model is generally greater than one, so it is not physically possible. Additionally, the effect of measurement noise will affect the observation precision. To solve this problem, the inverse of the model can be multiplied by a low-pass filter. According to the relative order of the nominal model above, we can design the low-pass filter as the form of the typical two order system, so that the relative order of the product by the inverse of the model and the low-pass filter will be equal to zero, thus avoiding the direct differentiation of the measured signal. The DOB after structural transformation is shown as shown in Figure 5.

Thus, after obtaining $\hat{\delta }$, the upper bound of $\Delta_{1}\Delta_{a}+\Delta_{2}$ can be determined approximately. Then the minimum switch gain simply needs to satisfy $\eta \geq \left|\Delta_{1}\Delta_{a}+\hat{\delta }\right|$ to guarantee the system stability and robustness. This means that the DOB can reduce the minimum switching gain and, thus, reduce the buffeting of the system on the premise of assurance of system robustness.

### 3.3. Rapid Nonlinear Tracking Differentiator

Note that the derivative of the signal is contained in the robust sliding mode control designed above. There are usually no corresponding sensors in the actual system. To obtain the approximate differential of a signal, a nonlinear tracking differentiator (NTD) is a good solution [25,26], as it makes use of the principle of signal tracking and guarantees the quality of the differential. Guo, B.-Z. and Zhao, Z.-L. give the general form of the NTD and gives a proof of it [31].

Suppose that the equilibrium point (0,0) of the following system is globally asymptotically stable:

(35) $$\left\{ \begin{array}{l} {\dot{x}}_{1}(t)=x_{2}(t),\text{ߓ}x_{1}(0)=x_{10}\\ {\dot{x}}_{2}(t)=f(x_{1}(t),x_{2}(t)),\;x_{2}(0)=x_{20} \end{array}\right.$$

among which $f:{\mathbb{R}}^{2}\to \mathbb{R}$ is a locally Lipschitz continuous function and f(0,0)=0. $x_{10}$ and $x_{20}$ are the initial value. If the differential needed signal r is differentiable and $\sup_{t\in [0,\infty ]}\left|\dot{r}(t)\right|<\infty$, then the following tracking differential:

(36) $$\left\{ \begin{array}{l} {\dot{z}}_{1R}(t)=z_{2R}(t),\;z_{1R}(0)=z_{10}\\ {\dot{z}}_{2R}(t)=R^{2}f[z_{1R}(t)-r(t),\;\frac{z_{2R}(t)}{R}]\;,\;z_{2R}(0)=z_{20} \end{array}\right.$$

is convergent in the sense that: for every a>0, $z_{1R}$ is uniformly convergent to r on [a,∞) as R→∞, where $z_{10}$ and $z_{20}$ are any given initial value. The selection nonlinear function f in (36) is a key problem which is also the focus of previous research.

However, [25] pointed out that there are always serious phase lags in the output no matter how the nonlinear function is designed. Thus, the NTD with feedforward is proposed to improve the rapidity:

(37) $$\left\{ \begin{array}{l} {\dot{z}}_{1R}(t)=z_{2R}(t),\;z_{1R}(0)=z_{10}\\ {\dot{z}}_{2R}(t)=R^{2}f[z_{1R}(t)-r(t),\;\frac{z_{2R}(t)}{R}]\;+k\dot{r}(t),\;z_{2R}(0)=z_{20} \end{array}\right.$$

where k (k>0) is a constant. A detailed proof is given in [25]. According to ([25], Remark 3.1), there is no need for $\dot{r}(t)$ in the implementation of the NTD, although the wanted signal differential $\dot{r}(t)$ appears in Equation (37).

In this paper, we make a slight improvement to Equation (37):

(38) $$\left\{ \begin{array}{l} {\dot{z}}_{1R}(t)=z_{2R}(t),\;z_{1R}(0)=z_{10}\\ {\dot{z}}_{2R}(t)=R^{2}f[z_{1R}(t)-r(t),\;\frac{z_{2R}(t)}{R}]\;+k\dot{r}(t)\times L(s),\;z_{2R}(0)=z_{20} \end{array}\right.$$

To reduce the impact of the noise of the input signal r(t), a low-pass filter L(s) is added to the feedforward. The cutoff frequency of the L(s) can be designed according to the specific requirements of the practical system.

**Figure 6:**
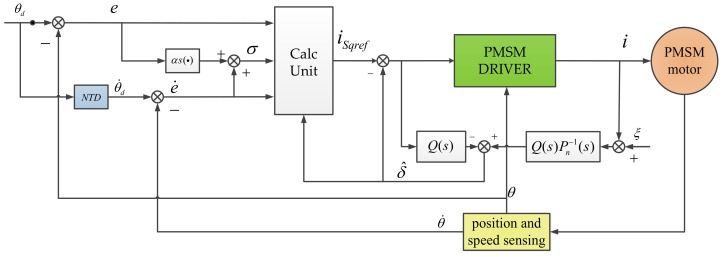
Robust sliding mode controller based on rapid NTD and DOB.

Thus, the block diagram of proposed robust sliding mode controller based on rapid NTD and DOB can be employed, as shown in Figure 6.

It is worth noting that the input signal of the NTD is acquired by adding e and θ, and the output signal of the NTD is ${\dot{\theta }}_{d}$. $\dot{e}$ is obtained by subtracting $\dot{\theta }$ from ${\dot{\theta }}_{d}$.

## 4. Implementation of Experimental System

**Figure 7:**
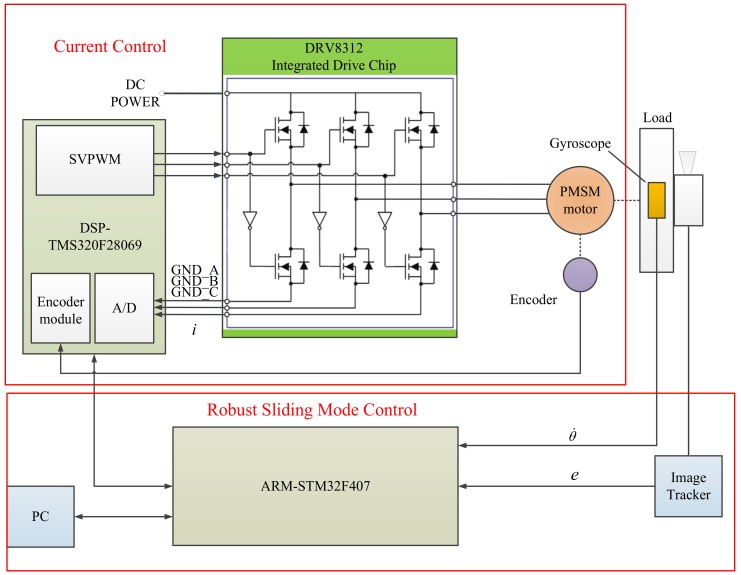
Configuration of the ARM and DSP-based experimental setup.

**Figure 8:**
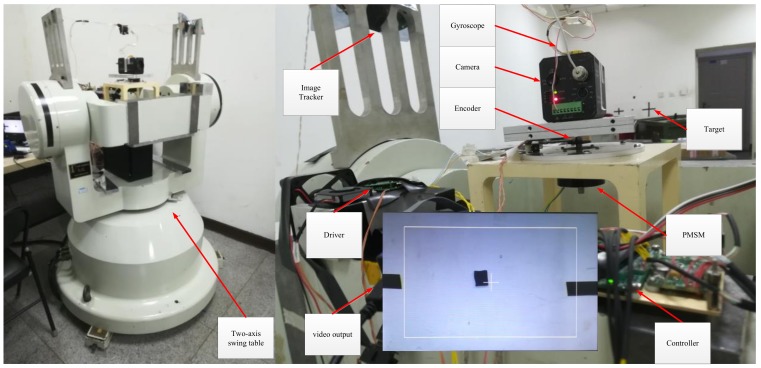
Photograph of the experimental platform.

The composition of the experimental system is shown in Figure 7, whereas Figure 8 shows the photograph of the experimental platform. The experimental system was mounted to a two-axis swing platform which was used to simulate the disturbance of the aircraft to the system. It is composed of a permanent magnet synchronous motor, a single-axis gyroscope, an off-axis encoder, driver, and acquisition circuit, control circuit, image tracker, visible light camera, and so on. The parameters of the tested PMSM is listed in Table 1. The high-precision single-axis gyroscope is employed for measurement of the angular velocity of the relative inertial space of the motor. In order to reduce the effect of friction on the shaft of the motor, an off-axis encoder was used. Unlike the photoelectric encoder, it works by using the principle of magnetic induction. The resolution ratio of the encoder is $360/2^{19}$ degrees and it is an absolute encoder. Angle detection is realized using the DSP (TMS320F28069) through the SPI interface and speed detection is realized using the ARM (STM32F407) through the RS422 serial port. The sampling frequency for the angle and current is 8 kHz, whereas the sampling frequency for the gyroscope is 2 kHz with a baud rate of 921.6 kbps. In this experimental system, DSP is mainly used to control the integrated drive chip DRV8312 in the current loop. The control strategy proposed in this paper is mainly implemented in ARM. The serial communication time of the DSP and the ARM is 1 ms. All programs are programmed in the C language.

The performance evaluation of the proposed robust sliding mode controller is presented in the following section.

## 5. Experimental Results and Discussions

To verify the effectiveness of the proposed robust SMC scheme in this paper, experiments are carried out under different strength sinusoidal disturbance conditions added by the swing platform, including d=3sin(2π×0.1t), d=6sin(2π×0.1t), d=sin(2πt), and d=sin(2π×2t) (unit: degrees). The following three methods are compared in the experiment: The traditional PI method, the robust SMC with differentiator using the Euler method, and the robust SMC with NTD. Based on the Z-N method [32], the parameters of traditional PI method are chosen as $k_{p}=0.2$, $k_{i}=1.0$; the parameters of the robust sliding mode controller are $k_{p}=0.1$, $k_{v}=1.0$, $k_{t}=0.6$, $\eta =5+\left|\hat{\delta }\right|$; ψ is determined to be 0.1 through multiple attempts; The parameters of the disturbance observer are:

(39) $$Q(s)=\frac{w_{p}^{2}}{s^{2}+2\beta_{p}w_{p}s+w_{p}^{2}}$$

where the cut-off frequency $w_{p}$ is set to be 6000rad/s, and the damping coefficient $\beta_{p}$ is set to be 0.7. The approximate current loop model P(s)≈1/(0.0001989s+1) is acquired by sweeping frequency.

The parameters of the rapid NTD are selected by referring to [25], the nonlinear function f is selected as:

(40) $$f(z_{1},z_{2})=-\alpha_{1}[{\left(\beta z_{1}\right)}^{\frac{p}{q}}+z_{1}]-\alpha_{2}[{\left(\beta z_{2}\right)}^{\frac{p}{q}}+z_{2}]$$

where $\alpha_{2}=2\alpha_{1}=2.0$, β=30.0, p/q=3, and k=650, and the low pass filter L(s) is in the same form with Q(s), but $w_{pl}=1256$ rad/s.

Additionally, the traditional PI method is adopted in the current loop of the PMSM driver, and the parameters of the three methods are all the same: d-axis PI parameters: $K_{dp}=8.0$ and $K_{di}=1.0$. q-axis PI parameters: $K_{qp}=8.0$ and $K_{qi}=1.0$. In order to compare the performance of the three control algorithms more fairly, the actual measurement is carried out when parameters are set, so that the measured bandwidth of the system is kept as consistent as possible, which is about 30 Hz (−3 dB).

In order to demonstrate the ability of the proposed method under different disturbance conditions, experiments are carried out in the tracking mode. By analyzing the data of the gyroscope, the inertial stability performance of the system can also be analyzed.

**Figure 9:**
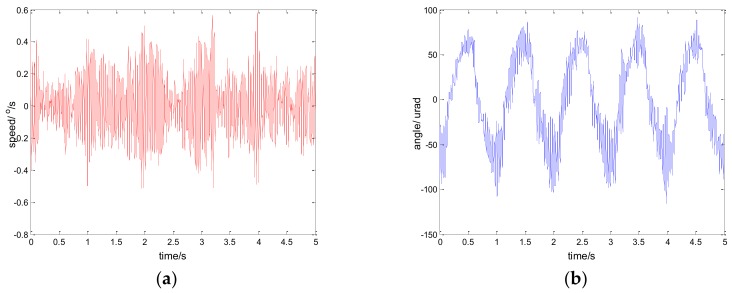
Experiment results of PI control. d=sin(2πt): (**a**) speed; and (**b**) tracking error, RMS = 48.6156 urad.

**Figure 10:**
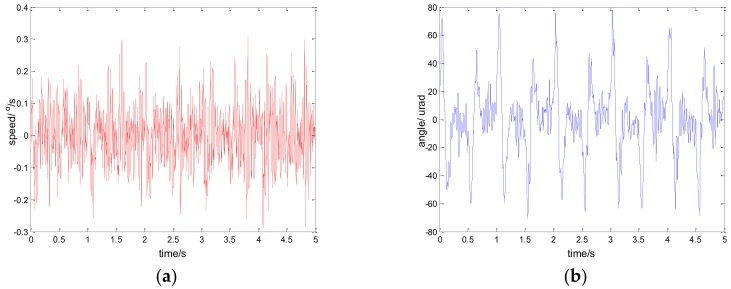
Experiment results of robust SMC with Euler. d=sin(2πt): (**a**) speed; and (**b**) tracking error, RMS = 25.7942 urad.

**Figure 11:**
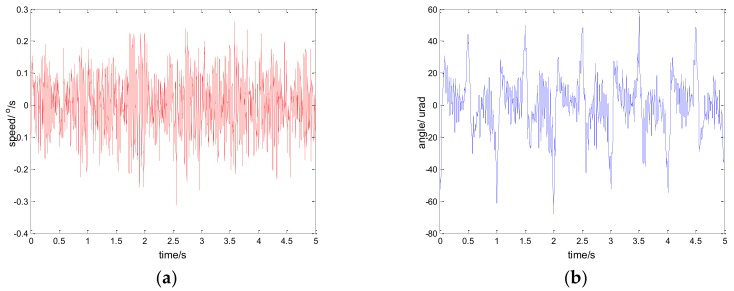
Experiment results of robust SMC with NTD. d=sin(2πt): (**a**) speed; and (**b**) tracking error, RMS = 17.5528 urad.

The experimental results of the PI, robust SMC with Euler, and robust SMC with NTD under the disturbance d=3sin(2π×0.1t) are shown in Figure 9, Figure 10 and Figure 11. Figure 9a, Figure 10a and Figure 11a show the speed signal of the gyro output, and Figure 9b, Figure 10b and Figure 11b show the tracking error obtained by integrating the speed signal. The tracking error shows the inertial tracking capability of the system. The RMS value of the tracking error is used for compared. The formula for calculating the RMS is:

(41) $$RMS={\left[\frac{1}{n-1}\sum_{i=1}^{n}{\left(x_{i}-\bar{x}\right)}^{2}\right]}^{\frac{1}{2}}$$

The experimental results demonstrate that, at the disturbance of sin(2πt), the robust sliding mode controller with NTD achieves a satisfactory inertial tracking performance. The tracking error RMS values of the three control methods are 48.6156 urad, 25.7942 urad, and 17.5528 urad, respectively. Compared to the PI method and the robust sliding mode controller with Euler, the tracking error RMS values of the robust sliding mode controller with NTD reduce by 63.89% and 31.95%, respectively. In order to verify the effectiveness of the proposed method under various disturbances, the following comparison experiments are also conducted.

**Figure 12:**
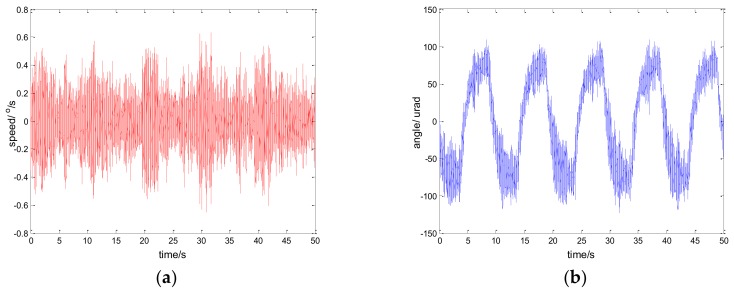
Experiment results of PI control. d=3sin(2π×0.1t): (**a**) speed; and (**b**) tracking error, RMS = 60.6991 urad.

**Figure 13:**
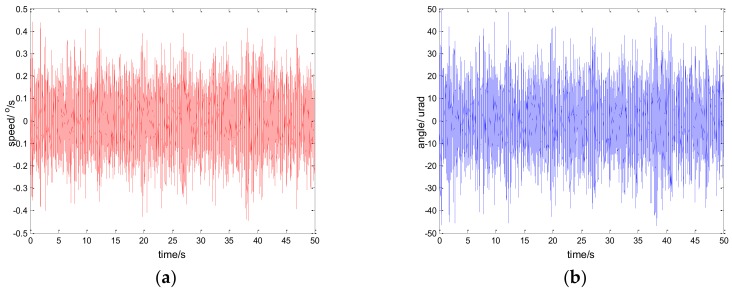
Experiment results of robust SMC with Euler. d=3sin(2π×0.1t): (**a**) speed; and (**b**) tracking error, RMS = 12.9125 urad.

**Figure 14:**
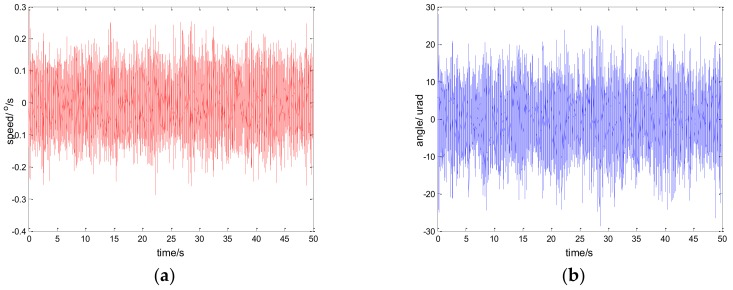
Experiment results of robust SMC with NTD. d=3sin(2π×0.1t): (**a**) speed; and (**b**) tracking error, RMS = 7.1381 urad.

**Figure 15:**
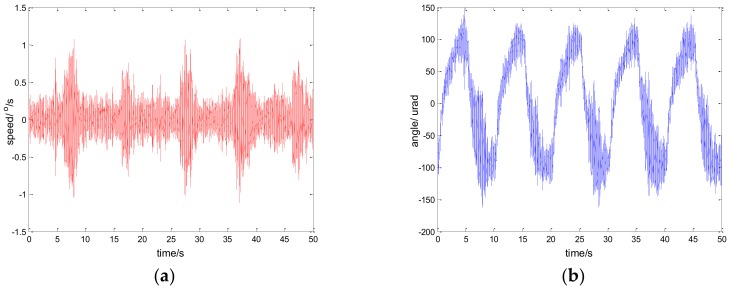
Experiment results of PI control. d=6sin(2π×0.1t): (**a**) speed; and (**b**) tracking error, RMS = 73.4717 urad.

**Figure 16:**
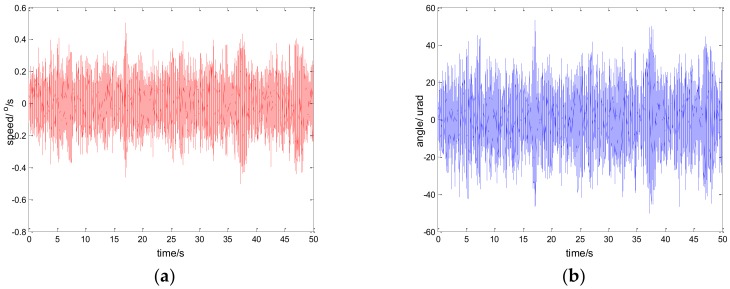
Experiment results of robust SMC with Euler. d=6sin(2π×0.1t): (**a**) speed; and (**b**) tracking error, RMS = 13.9755 urad.

**Figure 17:**
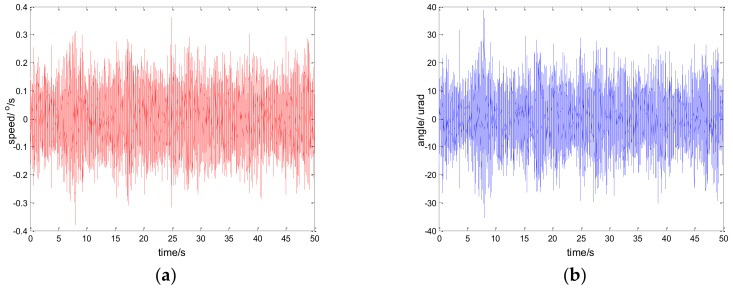
Experiment results of robust SMC with NTD. d=6sin(2π×0.1t): (**a**) speed; and (**b**) tracking error, RMS = 8.4473 urad.

**Figure 18:**
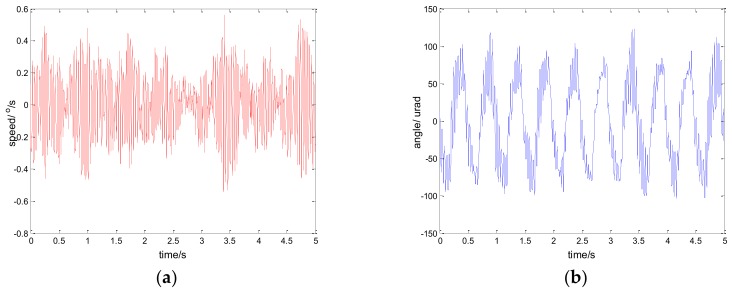
Experiment results of PI control. d=sin(2π×2t): (**a**) speed; and (**b**) tracking error, RMS = 55.7748 urad.

**Figure 19:**
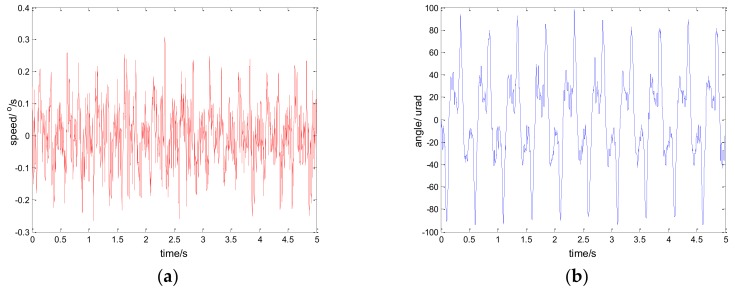
Experiment results of robust SMC with Euler. d=sin(2π×2t): (**a**) speed; and (**b**) tracking error, RMS = 39.5665 urad.

**Figure 20:**
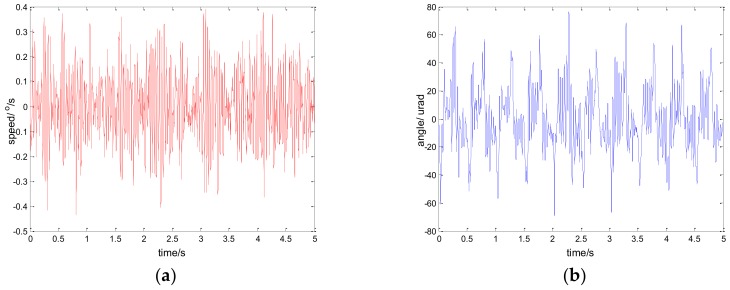
Experiment results of robust SMC with NTD. d=sin(2π×2t): (**a**) speed; and (**b**) tracking error, RMS = 23.0459 urad.

Figure 12, Figure 13 and Figure 14 show the experiment results in medium angle low frequency disturbance signal d=3sin(2π×0.1t) with the maximum acceleration of $1.18^{o}{/s}^{2}$; Figure 15, Figure 16 and Figure 17 show the experiment results in the large angle, low-frequency disturbance signal d=6sin(2π×0.1t) with the maximum acceleration of $2.37^{o}{/s}^{2}$; Figure 18, Figure 19 and Figure 20 show the experiment results in small angle, high-frequency disturbance signal d=sin(2π×2t) with the maximum acceleration of $157.91^{o}{/s}^{2}$. In addition, d=sin(2πt) is the small angle, medium-frequency disturbance signal with the maximum acceleration of $39.48^{o}{/s}^{2}$.

**Figure 21:**
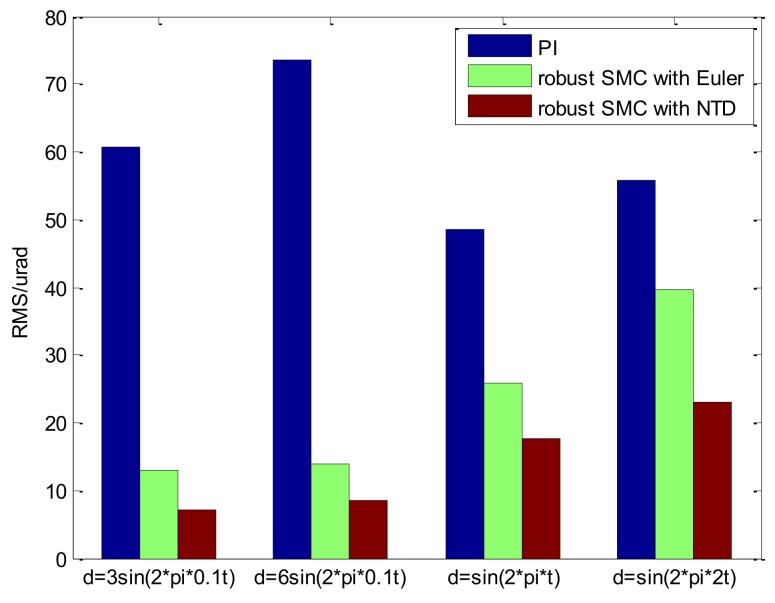
Comparison results of the tracking error in RMS.

From the experimental results presented, it is evident that the proposed robust SMC with NTD has obvious advantages. For a more intuitive comparison, the RMS value column diagram of the tracking error under different disturbance conditions is shown in Figure 21. What is worth paying attention to here is that when only the PI method is compared, the RMS value of the tracking error under the disturbance of 3sin(2π×0.1t) and 6sin(2π×0.1t) with small maximum acceleration is even higher than the others. This is due to the existence of friction and other disturbances in the system, and the other two methods, due to the use of the disturbance observer, this phenomenon is well suppressed.

**Figure 22:**
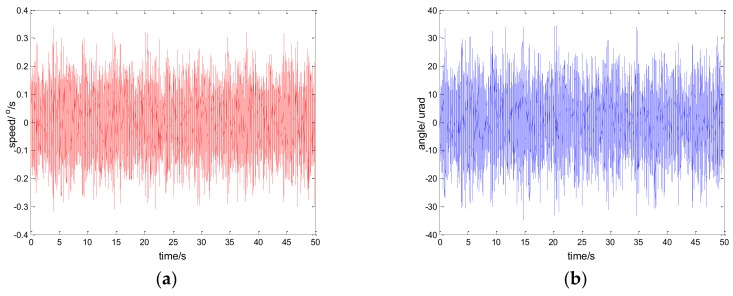
Robustness experiment results of robust SMC with NTD. d=3sin(2π×0.1t): (**a**) speed; and (**b**) tracking error, RMS = 9.9620 urad.

**Figure 23:**
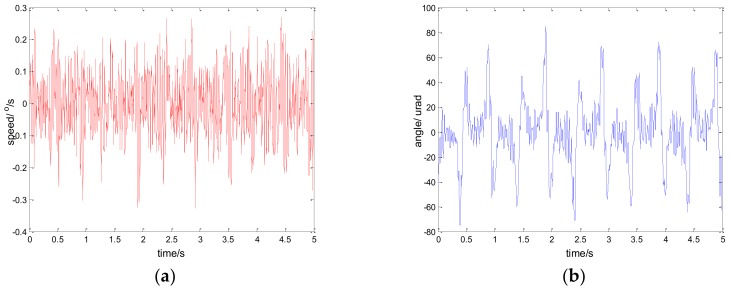
Robustness experiment results of robust SMC with NTD. d=sin(2πt): (**a**) speed; and (**b**) tracking error, RMS = 25.9620 urad.

Considering the robustness of the proposed method, we change the parameters of the controlled object by artificially increasing the load [33]. Specifically, we use the method of sticking lead blocks to achieve this. In fact, the inertia J is increased in Equation (13) in this way. By testing the performance of the system in this case, we can indirectly weigh the robust performance of the system when the model changes. Figure 22 shows the experiment results under the disturbance signal d=3sin(2π×0.1t) by adding a 50 g lead (the load weight of the original system is about 800 g); Figure 23 shows the experiment results under the disturbance signal d=sin(2πt) by adding an 80 g lead.

Comparing the results of Figure 23 and Figure 11, Figure 22 and Figure 14, respectively, we can determine that although the performance of the system is lower than that of the original, it still has good performance. To a certain extent, the robustness of the controller is verified. In practical applications, due to the influence of environment, such as temperature, humidity, mechanical wear, and so on, the parameters of the system will change. Therefore, the robustness of the system is of great significance.

## 6. Conclusions

A robust sliding mode control strategy based on a rapid nonlinear tracking differentiator and disturbance observer is presented in this paper, for the purpose of improving the anti-disturbance ability of the PMSM inertial tracking system, so to achieve better inertial stability. Sliding mode control is a reasonable choice for its invariance to the disturbance and the complexity of disturbance. The rapid nonlinear tracking differentiator provide the derivative for SMC controller. Disturbance observer is utilized to estimate the lumped disturbances of the system. The estimated disturbance is utilized to compensate the robust sliding mode controller, which can reduce the minimum switching gain and, thus, alleviate the system sliding mode chattering simultaneously. Experimental investigations were conducted on an integrated ARM-DSP-based PMSM platform. The PMSM servo system was operated under different kinds of sinusoidal disturbances coming from a two-axis rotating platform. The effectiveness of the proposed robust SMC with an NTD scheme was demonstrated by the experimental results.

## Figures and Tables

**Table 1:** Parameters of PMSM.

Description	Value
Peak torque	≥0.75N⋅M
Continuous plugging torque	≥0.15N⋅M
Rated voltage	24 V
Peak current	13.8 A
Armature resistance	11.8 Ω
Armature inductance	28 mH
Speed (Max. no-load)	3600 r/min
Number of pole pairs	4
Initia	0.5 $\mathrm{Kg}\cdot m^{2}\times 10^{-5}$
